# A Multi-Modal AI-Driven Cohort Selection Tool to Predict Suboptimal Non-Responders to Aflibercept Loading-Phase for Neovascular Age-Related Macular Degeneration: PRECISE Study Report 1

**DOI:** 10.3390/jcm12083013

**Published:** 2023-04-20

**Authors:** Michal Chorev, Jonas Haderlein, Shruti Chandra, Geeta Menon, Benjamin J. L. Burton, Ian Pearce, Martin McKibbin, Sridevi Thottarath, Eleni Karatsai, Swati Chandak, Ajay Kotagiri, James Talks, Anna Grabowska, Faruque Ghanchi, Richard Gale, Robin Hamilton, Bhavna Antony, Rahil Garnavi, Iven Mareels, Andrea Giani, Victor Chong, Sobha Sivaprasad

**Affiliations:** 1 Centre for Applied Research, IBM Australia, Southbank, VIC 3006, Australia; 2 National Institute of Health Research, Moorfields Biomedical Research Centre, Moorfields Eye Hospital, London EC1V 2PD, UK; 3 Frimley Health NHS Foundation Trust, Surrey GU16 7UJ, UK; 4 Department of Ophthalmology, James Paget University Hospitals NHS Foundation Trust, Norfolk NR31 6LA, UK; 5 Clinical Eye Research Centre, St. Paul’s Eye Unit, The Royal Liverpool and Broadgreen University Hospitals NHS Foundation Trust, Liverpool L7 8YE, UK; 6 Leeds Teaching Hospitals NHS Trust, Leeds LS1 3EX, UK; 7 South Tyneside and Sunderland NHS Foundation Trust, Sunderland SR4 7TP, UK; 8 Newcastle Hospitals NHS Foundation Trust, Newcastle upon Tyne NE1 4LP, UK; 9 King’s College Hospital NHS Foundation Trust, London SE5 9RS, UK; 10 Bradford Teaching Hospitals NHS Foundation Trust, Bradford BD9 6RJ, UK; 11 York Teaching Hospital NHS Foundation Trust, York YO31 8HE, UK; 12 Boehringer Ingelheim, 55218 Ingelheim am Rhein, Germany; 13 Institute of Ophthalmology, University College London, London NW3 2PF, UK

**Keywords:** age-related macular degeneration, predictive modelling, multimodal artificial intelligence, aflibercept, loading-phase

## Abstract

Patients diagnosed with exudative neovascular age-related macular degeneration are commonly treated with anti-vascular endothelial growth factor (anti-VEGF) agents. However, response to treatment is heterogeneous, without a clinical explanation. Predicting suboptimal response at baseline will enable more efficient clinical trial designs for novel, future interventions and facilitate individualised therapies. In this multicentre study, we trained a multi-modal artificial intelligence (AI) system to identify suboptimal responders to the loading-phase of the anti-VEGF agent aflibercept from baseline characteristics. We collected clinical features and optical coherence tomography scans from 1720 eyes of 1612 patients between 2019 and 2021. We evaluated our AI system as a patient selection method by emulating hypothetical clinical trials of different sizes based on our test set. Our method detected up to 57.6% more suboptimal responders than random selection, and up to 24.2% more than any alternative selection criteria tested. Applying this method to the entry process of candidates into randomised controlled trials may contribute to the success of such trials and further inform personalised care.

## 1. Introduction

Age-related macular degeneration (AMD) is the most common cause of visual impairment in the developed nations, affecting hundreds of millions of people worldwide [1,2]. Neovascular AMD (nAMD) is characterised by the presence of macular neovascularisation (MNV). Exudation from active MNV is visualised by optical coherence tomography (OCT) as presence of intra- and/or subretinal fluid. Anti-vascular endothelial growth factor (anti-VEGF) therapy is aimed at the resolution of macular fluid and is the current standard of care for exudative nAMD [3]. However, response to this treatment is heterogeneous [4,5,6,7].

Aflibercept is a commonly used anti-VEGF agent globally [8]. The loading-phase of aflibercept consists of a series of three intravitreal injections administered at a fixed monthly interval, and further re-treatment is guided mainly by the presence of macular fluid on OCT at approximately 8 weeks after the third injection [9]. The ability to identify suboptimal responders with persistent macular fluid post loading-phase based on their baseline characteristics may have implications for current and future patients. Presently, it would enable improved care planning for patients and clinicians alike, by identifying individuals who are likely to require earlier retreatment post loading-phase. In the long term, it would support the investigation of novel drugs with different mechanisms of action. Ex ante identification of early suboptimal responders could inform the design of randomised controlled trials (RCTs) of investigational agents by enriching the study cohort with treatment-naïve patients predicted to have a suboptimal response to aflibercept. This in turn will help in effective personalisation of treatment for this condition in the future.

Patient selection and suitability is one of the key challenges of RCT design, and incorrect patient selection can be detrimental to the success of a trial. Artificial intelligence (AI) systems can be used to aid patient selection for a clinical trial by using the patient’s predicted response to the trialled drug (predictive enrichment) and/or predicted clinical outcome (prognostic enrichment) [10,11,12].

In this first of a series of PRECISE study reports, we present and evaluate the efficacy of an AI-based cohort selection tool for the identification of patients for an enriched trial cohort based on their predicted suboptimal response to loading-phase aflibercept, defined as persistent macular fluid post loading-phase.

## 2. Materials and Methods

The study (registered ISRCTN28276860) was approved by the National Research Ethics Service in the UK (REC no: 19/LO/1385) and adhered to the Declaration of Helsinki. All participants provided written informed consent. Patients recruited retrospectively provided consent for their pseudo-anonymised data on the loading-phase to be used for the study, while the patients recruited prospectively provided consent for the assessments to be performed as per study protocol and for their pseudo-anonymised data to be used for the study.

### 2.1. Study Design

#### 2.1.1. Study Participants

**Figure 1:**
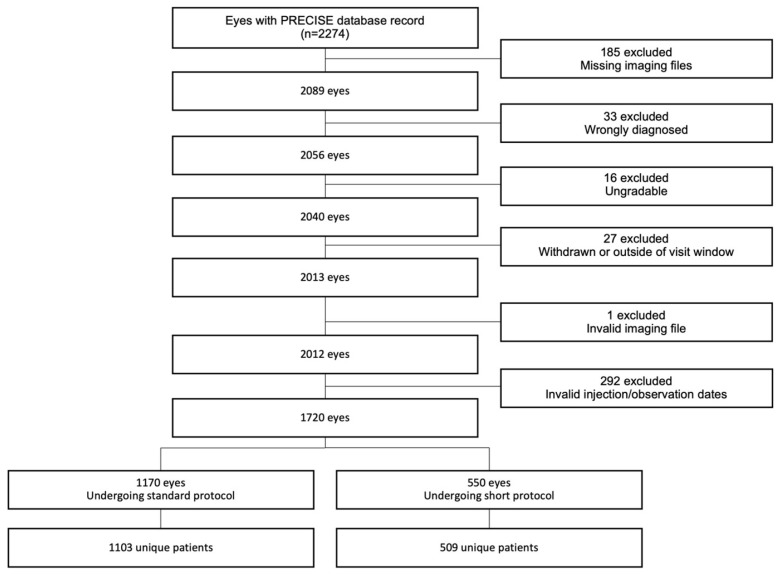
Participant flow.

The cohort was composed of treatment-naïve adults aged 50–100 years diagnosed with active MNV across 10 clinics in the UK. Therapy consisted of three intravitreal aflibercept injections with a follow-up within 10 weeks of the last injection. Patients were recruited between 18 December 2019 and 4 August 2021. The retrospective cohort included patients who initiated aflibercept therapy before the start date of the study, and the prospective cohort included patients who were diagnosed and initiated aflibercept after December 2019. Only those who had spectral domain OCT scans with Heidelberg Spectralis OCT (V6.15, Heidelberg Engineering GmbH, Heidelberg, Germany) at baseline and at follow-up visit post loading-phase were included. Other exclusion criteria were co-existent ocular conditions that, in the opinion of the local clinic’s investigator, could affect the visual acuity or macular fluid resolution, such as epiretinal membrane and vitreomacular traction. There were no visual acuity restrictions (Figure 1).

**Figure 2:**
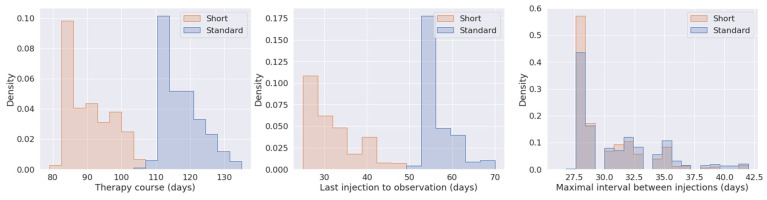
Temporal differences between eyes undergoing standard versus short protocol. Density shown on the x-axis refers to total count of eyes normalised to one.

As is often the case in real-world studies, there were some clinic- or patient-specific protocol variations. As such, we identified two main treatment protocols: eyes belonging to the standard protocol subcohort had a therapy course of 105–135 days, and about 8 weeks between third injection and final observation. Eyes belonging to the short protocol subcohort had a therapy course of 75–104 days, and about 4 weeks between last injection and final observation. There was no significant difference in injection interval between protocols (Figure 2).

#### 2.1.2. Study Assessments

All assessments and treatment procedures were performed as per standard of care. The OCT raster scan was centred at the fovea (range 20° × 25° to 30° × 20°) using 19, 25, 31, or 49 line scans. Each clinic employed one or more image acquisition protocols, which differed in resolution and the overall number of line scans as per the above. OCT scans were compulsory at baseline and final visit for study inclusion.

#### 2.1.3. Data Collection

Demographic data included age at presentation, sex, and ethnicity. Clinical data collected in a web-based database included visual acuity using standardised ETDRS (Early Treatment Diabetic Retinopathy Study) letter charts, central retinal subfield thickness (CST), total retinal volume (TRV), and dates of OCT and injections at all visits. The AI system used only features that were measured in the baseline exam, prior to therapy commencement. 

#### 2.1.4. Grading of OCT Images

The OCT images were graded at Moorfields Eye Hospital, London, UK, by four medical retina fellows with at least 5 years’ experience in grading OCT images (Sh.C., S.T., E.K., Sw.C.). All images were graded on hospital computers with 20-inch screens. The fellows were initially trained on a set of 50 OCT images and obtained a kappa [13] of 0.94 for inter-grader agreement on macular fluid assessment, which is considered a high level of agreement. Each scan was corrected for any segmentation errors and foveal centring before grading. Firstly, the baseline scans were verified for the presence of MNV based on the Consensus on Neovascular Age-Related Macular Degeneration Nomenclature (CONAN) [14] classification. Cases with no evidence of MNV were excluded (Figure 1) Subsequently, the macula scans at final follow-up were reviewed for the presence or absence of any residual macular fluid (intra- or subretinal fluid).

#### 2.1.5. Outcome Definition

A good response to aflibercept loading-phase (denoted ‘0’) was defined as total absence of both intra- and subretinal macular fluid in the macular volume scan at the final follow-up. A suboptimal response was defined as presence of any macular fluid (denoted ‘1’) at the final follow-up.

### 2.2. AI System Development 

#### 2.2.1. Data Splitting

To develop an AI-based treatment response selection, we divided all OCT scans into training, validation, and test sets. First, from the overall number of patients, 20% were randomly selected for test; 20% of the remaining patents were then selected for validation.

Splits were generated at the patient level to avoid having the same patient in the training and test sets. We stratified by protocol (standard or short), binary treatment outcome, and scan size. Due to the short protocol subcohort size, we subsequently merged all non-training samples of the short protocol into a single hold-out test set. All algorithms were thus only tuned to perform well on the validation set of the standard protocol or the training set of the short protocol (see the transfer learning step in the Supplementary Materials). Both subcohorts’ training sets were further split into five folds, again by randomly sampling patients, stratified by clinic and outcome.

#### 2.2.2. Pre-Processing

All OCT scans were first subjected to a standardised pre-processing algorithm. This consisted of three individual steps. First, the raw images were loaded scan-by-scan by their order in the volume and resized by linear interpolation to a unified height of 200 pixels and width of 300 pixels. Subsequently, we subsampled all individual OCT scans to yield a total of 20 scans per eye. This was done by selecting 20 scans from the total number of scans in regular intervals, for eyes with more than 20 scans, and evenly duplicating scans, for eyes with less than 20 scans. Lastly, we normalised each resulting 20 × 200 × 300 data matrix by mapping its individual 1% and 99% percentile to −1 and 1, respectively. We did not normalise by common reference due to the large variability in intensity between studies.

Clinical features were normalised as follows. For numerical features (e.g., age), we extracted the mean and standard deviation (SD) from the training set only to scale all values (including validation and test sets) to have a mean of 0 and SD of 1 by subtracting the mean and dividing by the SD of all training samples. After normalising, missing values were imputed by the mean (i.e., 0). CST was the only strictly positive variable that was strongly skewed, and we first transformed the natural scale of the variable by applying the natural logarithm followed by normalisation. Binary (sex) and multi-class variables (clinic) were one-hot encoded.

#### 2.2.3. Augmentation

Images were further pre-processed by implementing a two-dimensional (2D) wavelet decomposition via a Daubechies order 1 wavelet [15], to subsequently only use ‘edges’ as image input, again individually standardised to a mean of 0 and SD of 1. The size of the input matrix was not changed by the wavelet transformation. A simple on-off Boolean hyperparameter controlled whether models were trained purely on the original OCT scans or on their wavelet transformations.

We also introduced a simple foreground detection algorithm. We aimed to find both the upper and lower background of the relevant retinal layers by reviewing the OCT scans by image column, marking the start of tissue by the following criteria. Since there appeared to be a salient ‘edge’ between the retinal layers and the upper background, we based the detection on a composite criterion: seen from the top of the image, tissue detection by finding a significant ‘edge’ (based on the wavelet transformation) of at least two SDs above wavelet mean, logical-OR-connected with the surpassing of a baseline luminosity value, calculated by image column, regressed towards the luminosity of the whole image by 50%. For the lower background, as in general no distinct ‘edge’ was present, we introduced only a simple threshold value, calculated, again, based on the column-wise luminosity, regressed towards the luminosity of the whole image by 50%.

OCT image backgrounds were then set to 0 for all further training steps. Both upper and lower luminosity threshold values were controlled (multiplied) by hyperparameters pertaining to a respective luminosity percentile value, to enable coarser to finer background detection.

In addition to these image transformations, we applied image augmentations in every training epoch. These affine transformations were applied to each 2D scan, consistently for each OCT volume. The augmentations consisted of a rotation from −20° to +20°, translation of up to 10 pixels, flipping the image left to right, and scaling of the image, either zooming in, by 0 to 50%, or out, by 0 to 20%.

#### 2.2.4. Model Pipeline

The fuse-med-ml [16] (version 0.1.10, open-source Python) library was used throughout the image classification process. Given the binary labels, the ensuing classification task was attempted by three different model types, using the pre-processed OCT images or the clinical features as input.

First, we employed two different artificial neural network architectures: (a) a three-dimensional (3D) class activation maps network [17], and (b) a 2.5D Resnet50 network [18]. 

The former was a pure 3D convolutional neural network (i.e., 3D kernels) with six convolutional layers [19]. The latter consisted of a single 2D Resnet50 network [20,21] that was applied individually for every 2D scan (i.e., 2D kernels), followed by an averaging of output features from over all scans, which resulted in a total volume output (thus ‘2.5D’).

Both networks were adapted with an input layer to adjust for the size of OCT images (20-dimensional with only one channel each), as well as a classification layer based on the cross-entropy loss.

Hyperparameters included CNN type, batch size, learning rate, dropout rate, weight decay, background detection level, and wavelet transformation, as well as the training folds (1 out of 5 for cross-validation). Models were trained for up to 100 epochs using the Adam optimiser [22], after which we selected the best epoch on the cross-validation held-out set (1 out of 5). We ran hyperparameter tuning, randomly sampling parameters and choosing the best model based on its performance on the validation dataset. The final model was a 2.5D Resnet50 with input wavelet transformation, a batch size of 4, learning rate of 3.4 × 10^−6^, weight decay of 0.036, and dropout rate of 0.24.

Secondly, we trained a simple image classification model based on a 2D Fast Fourier Transformation (FFT) of every OCT scan. Given the sparsity of natural images in the Fourier domain, we first reduced the overall number of features by approximately 85% to the most dominant frequencies. The transformed one-dimensional vectors, representing the 2D frequency maps, were used as direct input for an l_1_-norm regularised logistic regression (Lasso [23]), with the volume labels applied to each scan. The strength of regularisation was tuned in 5-fold cross-validation. The final model reached in general a similar level of performance to the final CNN (see Results) but was dropped consecutively by the ensemble feature selection (see below).

Thirdly, a logistic regression with l_2_-norm regularisation based on all clinical features (i.e., age, sex, (log.) CST, visual acuity, and TRV) was standardised as described above. The strength of regularisation was again tuned in 5-fold cross-validation, after which the model was applied to the validation and test sets.

All models were either trained on standard or short protocol data, never on both simultaneously. However, since the performance of applying the CNN image classifier of the standard protocol subcohort on the short protocol subcohort was poor, we used the final CNN trained on standard protocol subcohort data to retrain on the short protocol subcohort tuning set, in order to employ the benefits of transfer learning [24,25]. Thus, we report three different kinds of model performances: models trained and tested on the standard protocol subcohort data, trained on standard data and tested on short protocol data (in general the least promising), and trained on short protocol data (CNN also pretrained on standard protocol subcohort data) and tested on short protocol data.

Finally, we trained an ensemble of all three model types to reach a single classifier. To this end, we used the outcome scores of all models [0–1] on the training data to also train an ensemble in the form of a logistic regression classifier with l_2_-norm regularisation, again tuned in 5-fold cross-validation. We ran a forward feature selection algorithm [26] for the standard protocol data, selecting the CNN as well as the clinical feature classifier based on the prediction performance on the validation data. Adding the FFT-based classifier did not further improve the performance on validation. This procedure was not repeated for the short protocol dataset.

### 2.3. Statistical Analysis

Univariate associations of clinical features and outcome were estimated using Fisher’s exact test for binary features and Mann–Whitney U test for continuous features. We corrected for multiple hypotheses by employing the Benjamini–Hochberg false discovery rate (FDR) correction. Area under the receiver operating characteristic curve (AUROC), sensitivity, and specificity are reported with a 95% confidence interval (CI). A *p*-value < 0.05 was considered to indicate a statistically significant difference. 

## 3. Results

The PRECISE study database includes records of 2274 eyes. The final dataset consisted of 1720 eyes of 1612 patients across 10 clinics (Figure 1). After recruitment and grading of the OCT scans, we excluded eyes that were misdiagnosed as MNV due to neovascular AMD, eyes with ungradable images, and eyes without available imaging files or outcome. Patients with missing or invalid dates of injections or observation were also excluded (Figure 1).

### 3.1. Study Population

We aimed to predict whether a patient’s eye would have residual retinal fluid (suboptimal response) post loading-phase therapy, given baseline OCT scan and clinical data. Our dataset consisted of 1720 eyes; however, careful examination of therapy course and intervals between injections and observation showed that two types of therapy protocols were in use, standard and short (Figure 2).

The standard protocol had a total follow-up duration of 105–135 days (16–17 weeks) from first injection, and last observation was 8 weeks after the third injection. The short protocol subcohort had a shorter follow-up, with the last observation taking place only 4 weeks post injections, resulting in a therapy course of 75–104 days (12–13 weeks). More eyes followed up on the short protocol showed complete macular dryness (χ^2^ test *p*-value < 4 × 10^−17^, odds ratio 2.4). Moreover, the use of the short protocol varied between clinics, but it has generally become more popular in recent years (Figures S1 and S2).

Moreover, the prospective cases in the PRECISE study were more recently enrolled so they tended to follow the short rather than the standard protocol (Figure S3). Consequently, all subsequent analyses were performed separately on the two subcohorts, based on protocol duration. The standard protocol subcohort comprised 1170 eyes of 1103 patients (mean age ± SD, 80 ± 8 years; Table 1 and Figure 1), while the short protocol subcohort comprised 550 eyes of 509 patients (79 ± 8 years). As the standard protocol subcohort represents the default treatment protocol, subsequent results focus on its results, while the short protocol subcohort results are described in the Supplementary Materials.

Older age was associated with good response to treatment (median age, 82 vs. 79 years, FDR adjusted *p*-value < 3 × 10^−11^; Table 2), as was female sex (67% vs. 59%, *p*-value < 4 × 10^−3^). CST was positively associated with suboptimal response (0.34 vs. 0.38, *p*-value < 1 × 10^−7^). Baseline absence or presence of fluid at the fovea was not associated with outcome (*p*-value > 0.05).

### 3.2. Treatment Response Prediction Using the AI System

The AI system is an ensemble of a machine-learning model, trained on clinical and ophthalmic features, and a deep-learning convolutional neural net (CNN), trained on OCT.

We split the standard protocol subcohort into 755 eyes (64%) in the training set, 187 eyes (16%) in the validation set, and 228 eyes (20%) in the held-out test set.

The AI system obtained an AUROC of 0.71 (95% CI 0.64, 0.78) in the standard protocol subcohort (Figure S4). The contribution of each model to the ensemble is summarised in Table 3. The clinical features that contributed the most to response prediction in the standard protocol subcohort were age, CST, and sex, in descending order (Figure S5). 

### 3.3. Comparison of Cohort Selection Methods

We emulated a candidate selection process for a hypothetical trial, preferring treatment-naïve suboptimal responders to loading-phase aflibercept. In different scenarios, this trial could consist of cohorts of different sizes. First, we used the AI system to prioritise patients based on their individual model scores (interpreted as the odds of being a suboptimal responder). Similarly, we prioritised patients based on baseline CST (descending order) and age (ascending order). Lastly, we added a random selection method to the comparison, which is the current clinical standard. For each hypothetical trial size between 20 and 120 eyes (drawn from the test data of each protocol), the AI-based selection method resulted in more suboptimal responders than any other method. It obtained an 18.6–57.6% increase in suboptimal responders compared with random selection. It also achieved a 9.4–24.2% increase compared with any other biomarker-based selection method, given different cohort sizes based on the standard protocol subcohort (see Table 4 for full comparison).

## 4. Discussion

In this study, we developed a clinical trial cohort selection tool based on predicted response to loading-phase aflibercept. Suboptimal response was defined as presence of macular fluid after the loading-phase of three monthly injections. A careful inspection of our dataset showed that the final treatment observation took place after heterogeneous periods of time (Figure 2). While the standard protocol states that follow-up should take place 8 weeks post injections, a shorter therapy protocol, with 4 weeks’ follow-up, has been in use to some extent, and by different clinics, for years. Use of the short protocol has become more prominent in recent years, especially in the UK during the COVID-19 pandemic (Figures S1 and S2). An eye undergoing short follow-up is 2.4 times more likely to show good response to treatment. The injection interval is similar for eyes undergoing standard and short protocols, so the difference in response is more likely to be a result of the shorter follow-up (Figure 2). In other words, patients who seem to be responding well at Week 4 may still relapse to suboptimal response at Week 8. The association between protocol duration and outcome, the association of certain clinics with short protocol use (Figure S2), and the use of different image acquisition protocols between clinics warranted a separate model for each protocol, and so all further analyses were performed separately on eyes that underwent either the standard or short protocol.

The univariate analysis showed a significant association between higher CST and worse response. This is to be expected given prior findings in this field [27]. Older age, on the other hand, was associated with better response. The reason for that is unclear but might be explained by the higher prevalence of retinal angiomatous proliferation in older individuals compared with the younger cohort [4]. Lastly, female sex was associated with better response, a correlation likely to be caused or strengthened by the significant association between older age and female sex. There was no association of baseline visual acuity or the existence of baseline retinal fluid in the entire macula with outcome. Although baseline visual acuity is a strong predictor of visual outcome at all milestones, this study reveals that it does not predict drying of macular fluid following loading-phase [4].

The AI system, based on minimal demographic and clinical features and non-annotated baseline OCT scans, demonstrated the potential of identifying patients’ anatomical response prior to loading-phase. It only used easily quantifiable standard clinical parameters that were previously identified to be associated with response to treatment [4,27]. Previous studies have used additional biomarkers and OCT parameters, such as location of fluid, to assess anatomical response to treatment and the need for more injections [28,29,30], as well as in the context of prediction of disease progression [31,32,33]. Bogunovic et al. attempted to predict patients’ pro re nata requirements based on their loading-phase OCT [29]. In addition, Schmidt-Erfurth et al. examined the correlation of baseline visual acuity and OCT features with visual acuity measured 12 months post treatment in the HARBOR trial and obtained an R^2^ of 0.34 [30]. However, our study aimed to predict the disease trajectory under a real-world non-singular protocol and non-annotated baseline OCT scans, a qualitatively different task, and may therefore not be compared with many previously reported results of AI systems on OCT tasks. The lack of association between baseline fluid and post-treatment presence of macular fluid further strengthens our hypothesis that being able to identify baseline fluid is not sufficient for an accurate anatomic response prediction.

The AI-based selection method performed better than other potential selection methods given the available data. We compared the ensuing selection based on predicted risk with two selection methods that are purely biomarker-based (age and CST), and also with random selection, which is likely to be the case in current randomised trials. Depending on the cohort size, the AI’s contribution to cohort selection varied, but was always advantageous in identifying suboptimal responders and better than the alternative methods.

Our study has some limitations. The AI system could be further improved by introducing annotations of the macular microstructures on OCT. For example, training the deep-learning model to identify intra- and subretinal regions, and especially fluid in those regions, could contribute both to the performance and explainability of our model. As a partial solution, we employed a classical denoising pre-processing stage that segments the region of interest. This may be improved in future work.

Another challenge faced in this study was the fovea location, which cannot be assumed to be the same for different scans. First, the use of different acquisition protocols meant that the fovea could appear in one or several line scans, depending on the OCT acquisition protocol and positioning of the grid during acquisition, and not necessarily the central scans in the volume. Secondly, the position and shape of the fovea in a certain scan could shift depending not only on the scan’s resolution but also, more importantly, on the disease state (e.g., presence of a large subfoveal pigment epithelial detachment or fluid distorting the shape and location of the fovea). In other words, our ability to identify (and subsequently grade) the fovea was confounded by the disease state. Due to this effect, we used global grading, considering fluid anywhere in the macular volume as suboptimal response to treatment.

Other studies have used visual acuity as an outcome instead of grading of macular fluid resolution [27,30,34]; however, basing response on macular fluid is more accurate to evaluate the efficacy of novel agents. Alternative therapies to aflibercept, such as faricimab [35,36], have recently been introduced to the market, targeting different pathways. Indeed, recently published randomised clinical trials that have examined alternatives to aflibercept have shown that while visual acuity did not improve further, the proportion of patients with residual retinal fluid decreased in comparison [35,37]. However, these trial designs do not permit the evaluation of novel agents in suboptimal responders to aflibercept.

The use of different OCT acquisition protocols, resulting in different resolutions as well as numbers and dimensionality of scan lines (even between eyes of the same patient), also means that each voxel may ultimately hold a different amount of information. Although not ideal from a modelling perspective, such a scenario is typical for real-world settings and, as a result, makes our model performance more indicative of how it may perform in a real-world clinical application. However, it may also result in a spurious association between acquisition protocol and outcome. We overcame this by extensive use of affine augmentations, as well as careful stratification in our training and validation datasets. 

Conversely, if our dataset was more homogeneous (i.e., having all eyes undergoing the same treatment guideline), we could have used its unprecedented size to the fullest. However, under current settings of real-world treatment protocols, we had to analyse the two subcohorts separately to avoid the spurious association between follow-up duration and outcome.

## 5. Conclusions

AI has the potential to accelerate scientific discoveries across all stages of development and validation of therapeutics. The candidate selection stage is vital for the success of a clinical trial and has implications for its cost and duration [10,38]. As such, there are some examples of how patient enrichment methodologies are being explored, particularly in oncology [11,39,40,41]. This study demonstrates how AI could identify optimal candidates based on their predicted response to the treatment available on the market. It may thus contribute to the success of future clinical trials for novel drugs and is likely to save time when treating patients. We believe this is the first of many solutions in other, wider, disease domains.

## Figures and Tables

**Table 1:** Patient characteristics for standard and short protocols.

	Standard Protocol	Short Protocol
No. of patients	1103	509
No. of eyes	1170	550
Therapy course, days *	117 ± 6	91 ± 6
Last injection to observation, days *	58 ± 3	32 ± 6
Age, years *	80 ± 8	79 ± 8
Sex: Female, *n* (%)	728 (62.2)	324 (58.9)
Ethnicity: White, *n* (%)	1070 (91.5)	515 (93.6)
South Asian, *n* (%)	6 (0.5)	10 (1.8)
Other, *n* (%)	94 (8)	25 (4.5)
Remaining retinal fluid post loading-phase	694 (59.3)	206 (37.5)

Unless otherwise indicated, *n* is number of eyes. * Data are mean ± standard deviation.

**Table 2:** Association of features of interest with therapy response for the standard protocol subcohort.

	No. of Eyes	Eyes with no Macular Fluid	Eyes with Macular Fluid	Adjusted *p*-Value
Age *	1170 (100)	82 [77, 87]	79 [73, 84]	2.97 × 10^−11^
Sex	1170 (100)	67%	59%	3.93 × 10^−3^
CST *	1169 (99.9)	0.34 [0.27, 0.43]	0.38 [0.31, 0.48]	1.23 × 10^−6^
Visual acuity *	1125 (96.2)	60 [50.00, 68.25]	60 [48.00, 70.00]	0.271

Data in parentheses are percentages. Median data and interquartile range. CST, central retinal subfield thickness. * Data are median [interquartile range].

**Table 3:** Performance summary of the AI system.

	Standard	Standard Applied to Short	Standard Tuned on Short
Logistic regression	0.63 [0.56, 0.71]	0.60 [0.55, 0.65]	0.64 [0.55, 0.72]
Convolutional neural network	0.70 [0.63, 0.77]	0.60 [0.55, 0.65]	0.67 [0.59, 0.75]
Final ensemble score	0.71 [0.64, 0.78]	0.62 [0.57, 0.66]	0.68 [0.61, 0.76]
90% sensitivity operating point			
Sensitivity	0.87 [0.81, 0.92]	0.86 [0.81, 0.91]	0.89 [0.82, 0.97]
Specificity	0.38 [0.29, 0.49]	0.23 [0.19, 0.28]	0.31 [0.23, 0.39]
90% specificity operating point			
Sensitivity	0.26 [0.17, 0.32]	0.26 [0.19, 0.31]	0.20 [0.16, 0.37]
Specificity	0.94 [0.88, 0.98]	0.85 [0.81, 0.88]	0.93 [0.85, 0.96]

The top three rows consist of AUROC values. Data in parentheses are 95% confidence intervals. AI, artificial intelligence; AUROC, area under the receiver operating characteristic curve.

**Table 4:** Fraction of suboptimal responders for the standard protocol subcohort in selected trial sizes.

Cohort Size (No. of Eyes)	AI	CST	Age	Random	% AI Increase	% AI Increase from Random
20	0.93	0.81	0.71	0.59	14.81	57.63
50	0.82	0.65	0.66	0.59	24.24	38.98
70	0.78	0.66	0.62	0.59	18.18	32.20
100	0.74	0.65	0.64	0.59	13.85	25.42
120	0.72	0.64	0.63	0.59	12.50	22.03
150	0.70	0.64	0.61	0.59	9.37	18.64

Candidates were selected from the held-out set of the standard protocol subcohort (228 patients). AI, artificial intelligence; CST, central retinal subfield thickness.

## Data Availability

The anonymised PRECISE clinical database analysed during the current study is available from author S.S. on approval of a data sharing agreement. Sharing of retinal images requires patient consent and sponsor approval.

## Supplementary Materials

The following supporting information can be downloaded at: https://www.mdpi.com/article/10.3390/jcm12083013/s1, Figure S1: Use of the standard versus short protocol as a function of time; Figure S2: Use of the standard versus short protocol across different clinics; Figure S3: (A) Number of prospective/retrospective cases as a function of time. (B) Prospective cases tend to follow the short protocol rather than the standard protocol; Figure S4: ROC curve and confusion matrices for the standard protocol subcohort; Figure S5: Feature contribution to the machine-learning model for the (A) standard and (B) short protocol subcohorts; Table S1: Association of features of interest with therapy response for the short protocol subcohort; Table S2: Fraction of suboptimal responders for the short protocol subcohort and selected sizes.
