# Supplementary material for: A Multi-Modal AI-Driven Cohort Selection Tool to Predict Suboptimal Non-Responders to Aflibercept Loading-Phase for Neovascular Age-Related Macular Degeneration: PRECISE Study Report 1
Source: J Clin Med. 2023 Apr 20;12(8):3013. doi: 10.3390/jcm12083013 (PMC10142969; doi:10.3390/jcm12083013)
Supplement: Supplementary file 1 [file jcm-12-03013-s001.zip › jcm-2185982-supplementary.pdf]

Supplementary Material

Results for the short protocol sub-cohort

Older age was associated with good response to treatment (median age, 81 vs 79 years, FDR adjusted p-value 0.048; Table S1). CST was positively associated with outcome (0.35 vs 0.4, p-value $<2\times10^{-3}$). Female sex was also significantly associated with good response to treatment (62% vs 54%, p-value $<6\times10^{-3}$). Baseline fovea dryness state was not associated with outcome (p-value >0.05).

To avoid overfitting due the short protocol sub-cohort's size, we split it only into tuning (359 eyes, 65%) and held-out test sets (191 eyes, 35%). Applying the standard protocol sub-cohort's ensemble model to the short protocol sub-cohort resulted in an AUROC of 0.62 (95% CI 0.61, 0.76). Transfer learning based on the standard protocol-based model on the tuning set of the short protocol sub-cohort resulted in an AUROC of 0.68 (95% CI 0.61, 0.76) (see Table 3 for breakdown). The clinical features that contributed the most to response prediction in the short protocol sub-cohort were TRV, sex, and CST (Figure S5).

When emulating a hypothetical candidate selection process based on the short protocol sub-cohort (each hypothetical trial size of 20–120 eyes drawn from the test data of each protocol), the AI-based selection method resulted in more suboptimal responders. It obtained a 16.2–75.7% increase in suboptimal responders compared with random and a 7.5–32.6% increase compared with the best performing alternative method (Table S2).

Supplementary figures

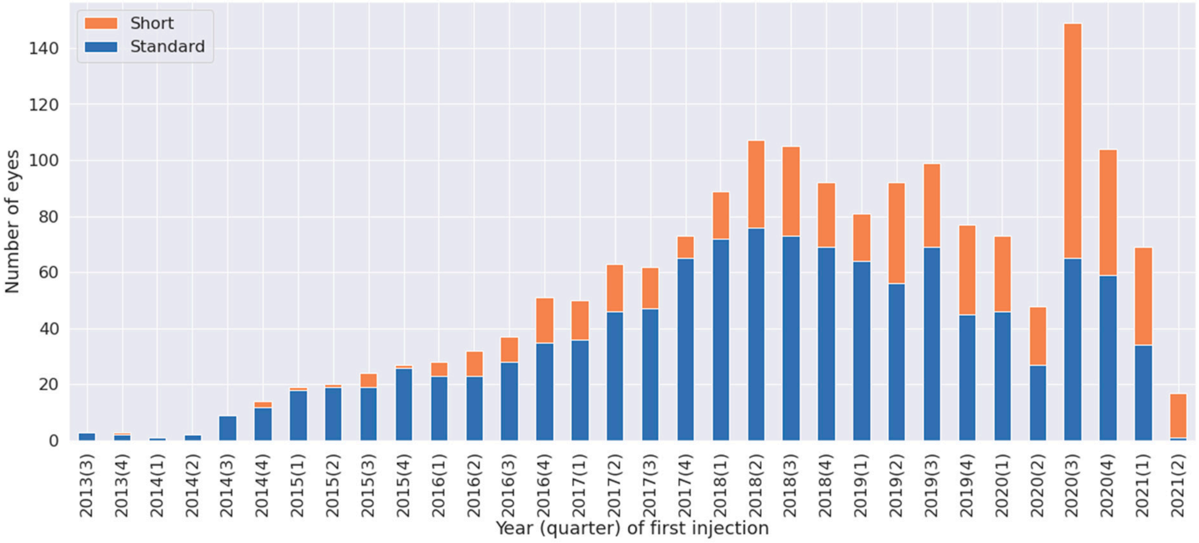

Figure S1. Use of the standard versus short protocol as a function of time

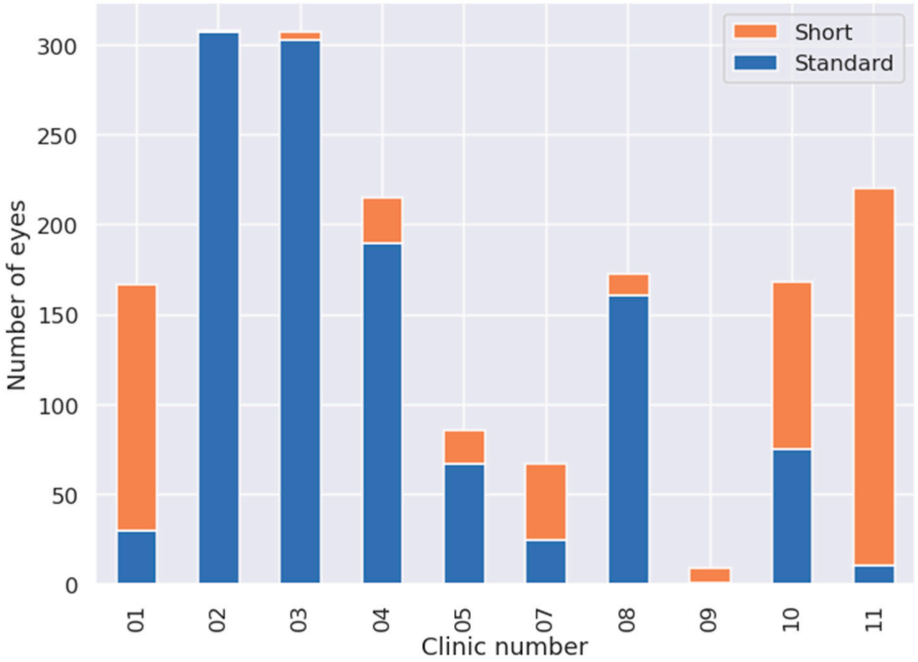

Figure S2. Use of the standard versus short protocol across different clinics

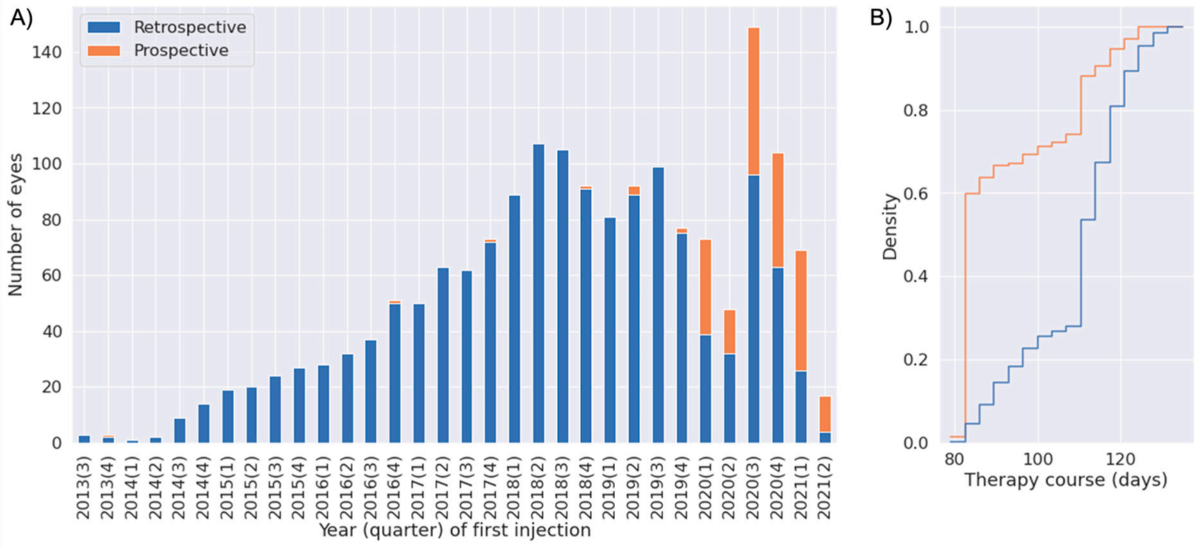

Figure S3. A) Number of prospective/retrospective cases as a function of time. B) Prospective cases tend to follow the short protocol rather than the standard protocol

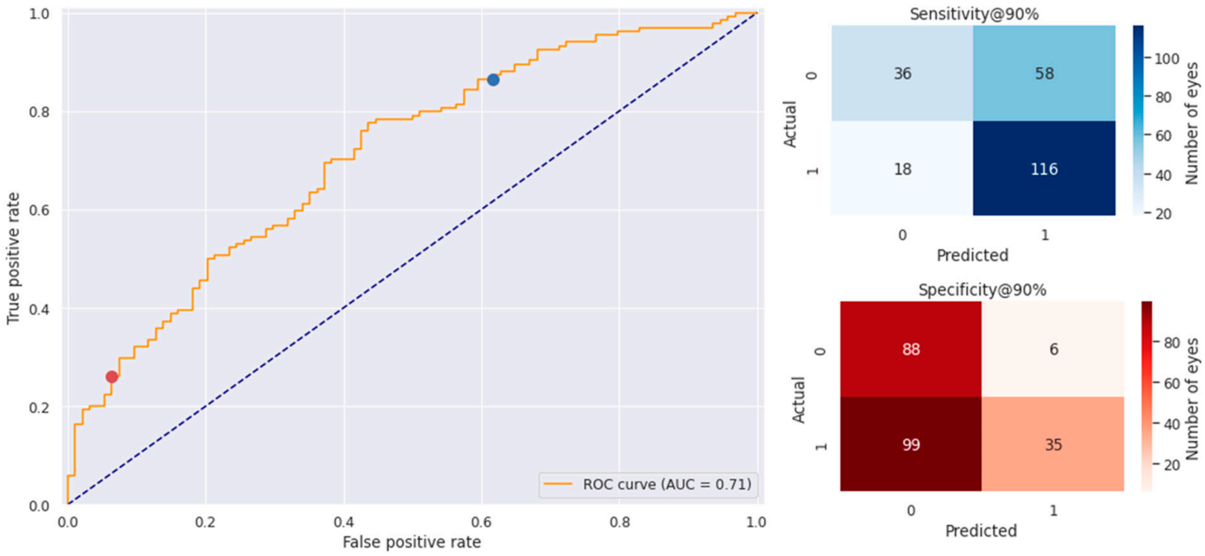

Figure S4. ROC curve and confusion matrices for the standard protocol sub-cohort

Blue dot represents 90% sensitivity. Red dot represents 90% specificity.

AUC, area under the curve; ROC, receiver operating curve.

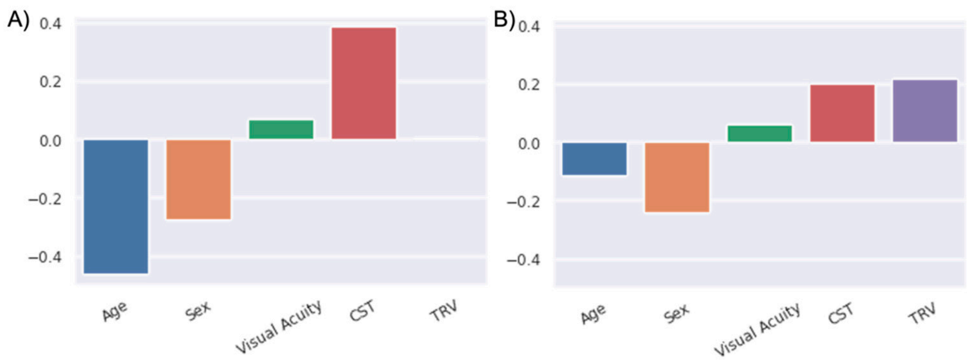

Figure S5. Feature contribution to the machine-learning model for the A) standard and B) short protocol sub-cohorts

Supplementary tables

Table S1. Association of features of interest with therapy response for the short protocol sub-cohort

	No. of eyes	Eyes with no macular fluid	Eyes with macular fluid	Adjusted p-value
**Age***	548 (99.6)	81 [75, 85]	79 [74, 83	$4.58{\times}10^{-2}$
**Sex**	550 (100)	62%	54%	$5.90{\times}10^{-3}$
**CST***	550 (100)	0.35 [0.29, 0.45]	0.4 [0.32, 0.51]	$2.06{\times}10^{-3}$
**Visual acuity***	536 (97.5)	60 [49, 70]	60 [47, 70]	0.729

Data in parentheses are percentages.

CST, central retinal subfield thickness.

*Data are median [interquartile range]

Table S2. Fraction of suboptimal responders for the short protocol sub-cohort and selected sizes

Cohort size (no. of eyes)	AI	CST	Age	Random	% AI increase	% AI increase

						**from random**
**20**	0.65	0.53	0.51	0.37	22.64	75.68
**50**	0.57	0.38	0.43	0.37	32.56	54.05
**70**	0.52	0.40	0.41	0.37	26.83	40.54
**100**	0.47	0.43	0.40	0.37	9.30	27.03
**120**	0.45	0.43	0.40	0.37	4.65	21.62
**150**	0.43	0.40	0.38	0.37	7.50	16.22

Candidates were selected from the held-out set of the short protocol sub-cohort (191 patients).

CST, central retinal subfield thickness.
